# The Exeter femoral stem continues to migrate during its first decade after implantation: 10–12 years of follow-up with radiostereometric analysis (RSA)

**DOI:** 10.3109/17453674.2012.672093

**Published:** 2012-04-24

**Authors:** Marc J Nieuwenhuijse, Edward R Valstar, Bart L Kaptein, Rob G H H Nelissen

**Affiliations:** ^1^Biomechanics and Imaging Group, Department of Orthopaedics, Leiden University Medical Center, Leiden; ^2^Department of Biomechanical Engineering, Faculty of Mechanical, Maritime, and Materials Engineering, Delft University of Technology, Delft, the Netherlands

## Abstract

**Background:**

Due to its collarless, double-tapered polished design, the Exeter femoral stem is known to migrate distally in the first 5 years after implantation. However, its long-term migration pattern has not been investigated.

**Patients and methods:**

39 consecutive patients (41 total hip arthroplasties) received a cemented Exeter stem and had prospective clinical and RSA follow-up. Patients were evaluated postoperatively at 6, 12, 26, and 52 weeks, and annually thereafter. Short-term results have been reported. In this study, the mean length of follow-up was 9.4 years (SD 3.2 years). No patients were lost to follow-up. 15 patients died during follow-up.

**Results:**

No stems were revised. In 4 stems, fractures of the cement mantle were noted within the first 3 postoperative years. In 3 stems, this resulted in a complete circumferential cement mantle discontinuity. For the 37 well-performing stems, continuous but small migration was measured between 2 and 12 years of follow-up. Continued subsidence of 0.08 mm/year (95% CI: 0.05–0.12, p < 0.001) was seen in combination with continued rotation in retroversion of 0.07°/year (95% CI: 0.02–0.12, p = 0.01). At 10 years of follow-up, mean subsidence was 2.1 (SD 1.2) mm and mean retroversion was 1.8° (SD 2.0). Two-thirds of this occurred during the first 2 postoperative years. In the 3 stems with a complete circumferential cement fracture, a sudden and disproportionately high increase in subsidence was measured in the time period of occurrence.

**Interpretation:**

The Exeter femoral stem continues to migrate during the first decade after implantation. Absolute stability is not required for good long-term survival if this is compatible with the design of the implant.

Due to its collarless, double-tapered polished design, the Exeter femoral stem facilitates distal migration within the cement mantle. Compared to other designs, the initial migration of the Exeter stem is high (Alfaro-Adrian et al. 1999, Kärrholm et al. 2000) and the stem continues to migrate up to 5 years after implantation (Stefansdottir et al. 2004). Its long-term migration pattern, however, is unknown but it has been claimed that the Exeter femoral stem continues to migrate during its entire lifespan (Carrington et al. 2009, Ling et al. 2009). This implies that the prosthesis never reaches a stable position. Nevertheless, the long-term survival of the Exeter stem is good and revision rates are low (Hook et al. 2006, Carrington et al. 2009, Havelin et al. 2009), which suggests that absolute stability may not be required for good survivorship of certain types of implants.

In addition, it has been shown that initial subsidence over 1.2 mm is associated with a high risk of future loosening of cemented Lubinus SP 1 stems (Kärrholm et al. 1994). However, the Exeter stem is designed to facilitate migration and this threshold may be inappropriate: high initial subsidence may not predict long-term failure of the Exeter stem (Huiskes et al. 1998, Alfaro-Adrian et al. 1999). A one-to-one relationship between initial migration and long-term survivorship can only be assessed by long-term RSA studies, since both the initial migration and the long-term outcome are known for the same patient. Currently, no long-term RSA studies evaluating this relationship have been published regarding the Exeter femoral stem.

In this prospective, long-term clinical and RSA follow-up study of 41 Exeter femoral stems, we analyzed the long-term migration pattern of this stem and evaluated its relationship to the (long-term) clinical outcome.

## Patients and methods

The patients in a previously reported, prospective, randomized double-blind study (Nelissen et al. 2005) on the short-term migration of the Exeter total hip arthroplasty (THA) fixated with a high- or a low-viscosity bone cement (Simplex AF and Simplex P, respectively; Stryker-Howmedica Inc., Kalamazoo, MI) remained under clinical and RSA follow-up in order to assess the long-term migration pattern and clinical outcome of the Exeter THA. In the present study we concentrated on the femoral component.

Between February 1997 and October 1998, 39 patients (mean age 70 (SD 6) years, 33 females) with 41 consecutive primary cemented THAs were included in the (original) study. 19 hips were operated for primary osteoarthritis and 22 for secondary osteoarthritis (20 rheumatoid arthritis and 2 ankylosing spondylitis). The implant used in all patients was the Exeter Universal THA, which consists of a collarless, double-tapered polished femoral stem and an ultra-high-molecular-weight all-polyethylene acetabular cup (Stryker-Howmedica Inc). Approval was obtained from the institutional review board and all patients gave written informed consent.

All patients were operated through a lateral approach in the lateral decubital position. Stem size 1 was used in 25 hips, stem size 2 in 15 hips, and stem size 3 in 1 hip; all stems had a 37.5-mm offset. For RSA measurements, 6–8 tantalum balls (1-mm) were inserted into the greater and lesser trochanter region during surgery. Furthermore, the implant manufacturer attached markers to the tip and shoulder of the stem. Patients were kept from weight bearing until the first RSA radiographs were taken (on the first or second postoperative day) and they were then allowed full weight bearing.

Patients were evaluated preoperatively and postoperatively at 6 weeks, 3 months, 6 months, 1 year, and annually thereafter. At each evaluation, the Harris hip score and RSA radiographs were obtained. Conventional anteroposterior and lateral radiographs were acquired at 6 weeks, 2 years, 5 years, and 10 years postoperatively and on indication (e.g. pain or suspected failure).

The fate of all 41 prostheses was known, and no patients were lost to follow-up. Mean length of follow-up was 9.4 (3.1–12.0) years. Follow-up of at least 10 years was available for 24 patients (26 prostheses); the 15 other patients died during follow-up due to causes unrelated to the THA.

The femoral component was considered a radiographic failure in the presence of cement cracks, complete progressive radiolucency of 2 mm or more, or fracture of the stem (Gruen et al. 1979, Johnston et al. 1990). Due to its design, initial subsidence of the stem was not considered to be indicative of failure (Gie and Ling 1994). Progressive continuous migration was considered to be indicative of failure, however.

RSA radiographs were obtained using a uniplanar setup with the patient in supine position and the calibration cage under the examination table. In 2002, the calibration cage was changed from the large reference box Leiden to the Carbon box Leiden. In 2004, the conventional radiography system was replaced by a digital radiography system. Both changes had no effect on the accuracy of the RSA measurements. Using the markers attached to the stem and the femoral head, a marker-based analysis was carried out (MB-RSA software; Medis Specials, Leiden, the Netherlands). The first RSA examination served as the baseline reference for all further examinations, and all evaluations are related to the relative position of the prosthesis to the bone at that time. Migration is expressed along or around the three orthogonal axes: longitudinal, transverse, and sagittal. The precision of RSA measurements was determined using 20 double examinations at the 1-year follow-up (Ranstam et al. 2000, Valstar et al. 2005) (Table 1).

**Table 1.** Precision of RSA measurements (upper limits of 95% confidence interval)

Stem	Transverse (x-axis)	Longitudinal (y-axis)	Sagittal (z-axis)
Translation (mm)	0.31	0.26	0.55
Rotation (degrees)	0.45	0.76	0.34

Mean condition number of the markers on the stem and in the femur was 8.7 (SD 0.7) and 28 (SD 21). Mean rigid body error in the analysis of the markers of the stem and femur was 0.15 mm (SD 0.07) and 0.21 mm (SD 0.09). These values satisfy the criteria in the guidelines by Valstar et al. (2005). 17 pairs of RSA radiographs in 5 patients had to be excluded due to technical problems.

On the 6-week postoperative standard anteroposterior and lateral radiographs, the stem orientation (i.e. varus or valgus) and cement mantle thickness were measured. Minimal, maximal, and average cement mantle thicknesses were measured in all 14 Gruen zones. The femur-stem index was determined and the quality of cement penetration was scored (Barrack et al. 1992). Mean stem orientation was 1.6° (SD 2.4) varus; mean minimal, maximal, and average cement thicknesses were 3.4 (SD 0.6), 5.9 (SD 1.0), and 4.6 (SD 0.7) mm. The mean stem-femur index was 0.39 (SD 0.04). Type-A cement penetration was noted in 20 stems and type-B cement penetration in 21 stems. There was no type-C cement penetration.

### Statistics

Measured values are reported as mean with standard deviation (SD) and range. Model estimates are reported as mean with 95% confidence interval (CI). To account for the longitudinal nature of the data and repeated measurements in the same patient, analysis of migration was carried out using linear mixed-model analysis (allowing random intercepts and random slopes). The steady-state migration rate was estimated using the regression coefficient of the mixed-model analysis. All available examinations were included in the analysis and the mean at every follow-up occasion represents the mean migration of all available prostheses (patients). Degenerative or inflammatory disease; stem size and orientation; cement type; minimal, maximal, and average cement mantle thickness; and cementing grade were successive model factors in analysis of migration.

Any p-value of less than 0.05 was considered significant. For significance of model factors, a Bonferroni correction was applied and a p-value of less than 0.01 was required (SPSS software version 16.0).

## Results

### Clinical results

The Harris hip score increased from 31 (SD 19) points preoperatively to 57 (SD 18), 71 (SD 20), 76 (SD 13), and 73 (SD 23) points at 6 weeks and at 1, 5, and 10 years postoperatively (p = 0.002).

**Figure 1.**
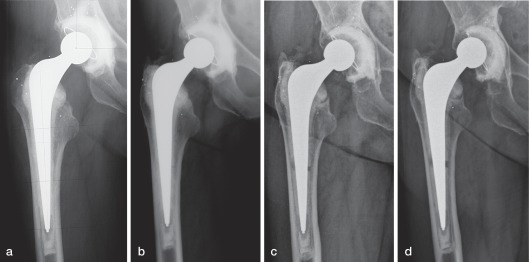
Patient 3 with a complete circumferential cement mantle fracture. a) 6 weeks post-operative radiograph: No abnormalities were seen. b) 2 year follow-up radiograph: A cement mantle fracture in Gruen zone IV is noted. c) 5 year follow-up radiograph: A complete circumferential cement mantle fracture is present. d) 10 year follow-up radiograph: Increased separation of the cement mantle. Between the 2 and 3 year RSA examination, a sudden and substantial increase in subsidence and retroversion of the stem was measured. At 12 year follow-up, subsidence was 9.7 mm and retroversion 11°.

No stems were revised during follow-up. However, in 4 stems (4 patients), fractures of the cement mantle were noted within 3 years (Table 2); 3 of these 4 patients died within 4 years. In 3 of these 4 patients, the cement mantle fractures resulted in a complete circumferential discontinuity of the cement mantle. One of these patients with a complete circumferential cement mantle fracture was available for long-term follow-up and had the only clinical stem failure in this study (Figure 1); progressive pain and deterioration of function started 5 years after occurrence of the cement mantle fractures. However, the patient refused revision.

**Table 2.** Details of patients with a failed Exeter stem

Patient	Primary diagnosis	Cement crack in Gruen zone	Cement viscosity	Time interval	Accompanying predominant migration	Years of follow-up	Reason
1	Osteoarthritis	II, VI, IX, XIII	low	3–6 months	Subsidence: 2.3 mm **^b^** Lateral translation: 0.6 mm	2	Died
2	Rheumatoid arthrits	I	high	1–2 years	Rotation (all 3 axes): 0.7–11°	3	Died
3	Osteoarthritis	II, VI, IX, XIII	low	2–3 years	Subsidence: 1.8 mm Retroversion: 3.5°	12	Alive
4	Ankylosing spondylitis	I, VI, XIII **^a^**	low	2–3 years	Subsidence: 1.0 mm **^b^**	3	Died

**^a^** In patient 4, there was incomplete cementing of zone II and IX. In patients 1, 3, and 4, there was a complete circumferential discontinuity in the cement mantle.

**^b^** No rotations could be measured for these patients (patient 1: only patient with no prosthesis marker on the shoulder; patient 4: insufficient suitable femur markers at follow-up).

### RSA results

**Figure 2.**
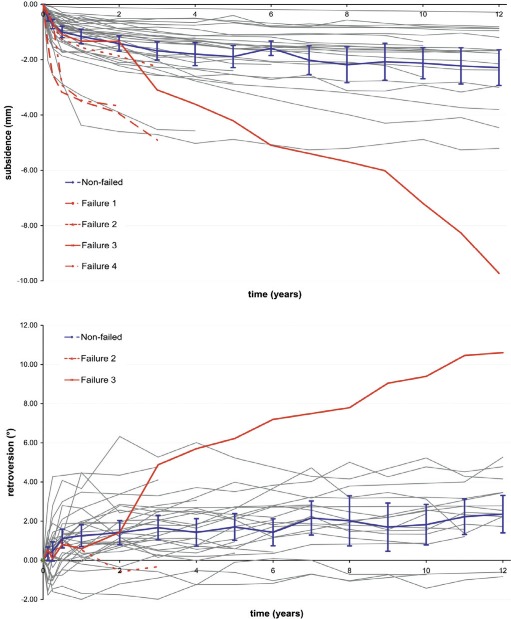
Subsidence (upper) and rotation in retroversion (lower) of the Exeter stems without cement mantle fractures (mean and 95% confidence interval, blue line) and of the individual failed components (red lines). The estimated yearly increase (slope of regression line) between 2 and 12 years was respectively 0.08 mm/year (95%CI: 0.05 – 0.12) and 0.07°/year (95%CI: 0.02 – 0.12). Failures 1, 3 and 4 had a complete circumferential cement mantle fracture. Solid grey lines represent migration of individual non-failed components.

In all 3 patients with a complete circumferential discontinuity of the cement mantle, the occurrence of fractures in the cement mantle was accompanied by a considerable and disproportionate increase in subsidence (2.3, 1.8, and 1.0 mm) (Figure 2). In 2 of these patients, rotations could not be determined (Table 2). In the third patient, sudden movement of 3.5° into retroversion was measured and both subsidence and rotation into retroversion continued over the remaining follow-up period (Figure 2).

It is noteworthy that in the period in which the cement mantle fracture occurred, the measured increase in subsidence was the largest in the corresponding period and disproportionally higher compared to the well-performing stems. One complete circumferential cement mantle fracture occurred in the follow-up period 3–6 months. Over this period, an increase in subsidence of 2.3 mm was measured for this stem compared to the other stems, which had a mean increase in subsidence of 0.27 mm (SD 0.25) (range –0.25 to 1.32). Over the period 2–3 years of follow-up, an increase in subsidence of 1.0 and 1.8 mm was measured in 2 stems with a complete circumferential cement mantle fracture, compared to the well-performing stems which had a mean increase in subsidence of 0.17 mm (SD 0.16) (range –0.09 to 0.62).

In the 37 femoral stems without cement mantle fractures, translation was measured in the cranial-caudal direction (subsidence, p < 0.001) combined with rotation along the longitudinal axis (retroversion, p < 0.001) and sagittal axis (varus, p = 0.02) in the first 2 postoperative years. At 2 years, mean subsidence was 1.42 mm (SD 0.82) (0.43 – 3.91), mean rotation into retroversion was 1.42° (SD 1.66) (–1.76 to 6.33), and mean rotation into varus was 0.24° (SD 0.74) (–1.46 to 2.63).

During the follow-up period of 2–12 years, continuous but small migration was measured (Figure 2 and Table 3). Continued subsidence of 0.08 mm/year (95% CI: 0.05–0.12; p < 0.001) was seen in combination with continued rotation in retroversion of 0.07°/year (95% CI: 0.02–0.12; p = 0.01). Translation in the medial-lateral and anterior-posterior direction stabilized, as did rotation around the transverse and sagittal axes. At 10 years of follow-up, mean subsidence was 2.13 mm (SD 1.15) (0.79–4.88) and mean retroversion was 1.82° (SD 1.98) (–1.42 to 5.23).

**Table 3.** Migration of the Exeter femoral stem and estimated annual rate over 2–12 years of follow-up of 37 femoral stems without cement mantle fractures

	Transverse	Translation (mm) Longitudinal	Sagittal	Transverse	Rotation (degrees) Longitudinal	Sagittal
2 years, mean [SD] range	0.01 [0.39] (–1.25 to 0.90)	–1.42 [0.82] (–3.91 to –0.43)	–0.05 [0.51] (–0.92 to 1.14)	0.13 [0.63] (–0.84 to 1.86)	1.42 [1.66] (–1.76 to 6.33)	–0.24 [0.74] (–2.63 to 1.46)
5 years, mean [SD] range	0.12 [0.45] (–0.85 to 0.96)	–1.89 [0.98] (–4.88 to – 0.40)	–0.18 [0.55] (–1.09 to 0.88)	0.04 [0.59] (–0.72 to 1.23)	1.70 [1.56] (–1.24 to 5.23)	–0.18 [0.61] (–1.42 to 1.16)
10 years, mean [SD] range	0.15 [0.58] (–0.60 to 1.81)	–2.13 [1.15] (–4.88 to –0.79)	–0.26 [0.56] (–1.08 to 0.92)	–0.04 [0.95] (–2.30 to 1.27)	1.82 [1.98] (–1.42 to 5.23)	–0.10 [0.73] (–1.31 to 1.63)
2- to 12-year rate (95%CI)	–0.01 (–0.03 –0.01)	–0.08 (–0.12 –0.05)	0.00 (–0.02–0.02)	–0.02 (–0.04–0.01)	0.07 (0.02–0.12)	–0.00 (–0.02 –0.01)
p-value	0.3	< 0.001	1.0	0.2	0.01	0.6

Measured values are presented as mean, standard deviation, and range; estimates are presented as mean and 95% confidence interval (95% CI).

Cement type (viscosity); maximum, minimum, or mean cement mantle thickness; cementing grade; degenerative or inflammatory disease; stem size; neck size; or orientation had no (statistically significant) influence on migration (pattern) of the stem (all p > 0.01). In particular, migration was similar whether high or low viscosity cement was used (Table 4). 

**Table 4.** Mean difference in migration^a^ a over 2–12 years of follow-up between stems cemented with high- or low-viscosity cement (37 femoral stems without cement mantle fractures)

High-viscosity vs. low-viscosity cement	Transverse	Translation (mm) Longitudinal	Sagittal	Transverse	Rotation (degrees) Longitudinal	Sagittal
Mean difference	–0.19	0.14	0.25	0.08	–0.73	0.38
(95% CI)	(–0.48 to 0.10)	(–0.43 to 0.70)	(–0.16 to 0.65)	(–0.45 to 0.62)	(–1.90 to 0.45)	(–0.09 to 0.84)
p-value	0.2	0.6	0.2	0.8	0.2	0.1

**^a^** Mean difference was independent of time for all 6 degrees of freedom: p = 0.7, p = 0.7, p = 0.6, p = 0.3, p = 0.6, p = 0.3.

## Discussion

We found that the Exeter femoral stem continues to migrate during the entire first decade after implantation. After the initial period of 2 years, in which around two-thirds of the total migration was measured, the stem continued to subside and rotate into retroversion during the following 10 years.

The amount of subsidence we measured is in agreement with reported values at short-term (2-year) follow-up of 1.13, 1.25, and 1.34 mm (Glyn-Jones et al. 2003, Stefansdottir et al. 2004, McCalden et al. 2010) and at medium-term (5-year) follow-up of 1.77 mm (Stefansdottir et al. 2004). Also, continued rotation into retroversion was reported at medium-term follow-up and the reported value of 1.6° (Stefansdottir et al. 2004) agrees well with our results (1.7°).

It is interesting to note that this pattern of continuous migration of the femoral component (subsidence and retroversion) has been considered to be a pattern of failure (Kärrholm et al. 2000, Hauptfleisch et al. 2006, Hook et al. 2006). However, the magnitude of the estimated annual migration was very small and we do not consider this migration to be clinically worrisome.

Our study demonstrates that absolute stability is not required for good long-term outcome. Based on our results combined with the results of long-term follow-up studies (Hook et al. 2006, Carrington et al. 2009) and reports from the registries (Espehaug et al. 2009, Havelin et al. 2009), continuous migration appears to be compatible with good long-term survivorship for this (polished, double-tapered, collarless) prosthesis design. However, we do not expect that this finding can be generalized to all femoral stems. Migration should be in accordance with the stem design, and for prostheses that are not designed to subside, continuous migration may indicate detrimental outcome (Kärrholm et al. 1994, Hauptfleisch et al. 2006).

It has been questioned whether the extent of initial migration measured can predict later failure of the Exeter stem, or more generally, of (polished, tapered, collarless) implants that are designed to migrate (Huiskes et al. 1998, Alfaro-Adrian et al. 1999, Stefansdottir et al. 2004, McCalden et al. 2010). Since the mean subsidence of the 37 implants with good long-term outcome was 1.4 mm at 2 years of follow-up, a higher migration threshold than 1.2 mm as proposed by Karrholm et al. (1994) for another design of cemented stems, is more appropriate for (polished, tapered, collarless) implants designed to migrate. In the present study, however, we could not determine an appropriate threshold. The Exeter stem has excellent reported clinical survivorship, and the design of the implant is therefore good; consequently, no migration pattern indicative of a flawed design could be detected.

All 4 radiographic failures in our series were due to fractures in the cement mantle. Whether a cement mantle fracture leads to failure of the implant may depend on the location and extent of the fracture. Using RSA, fractures of the cement mantle can be detected but they cannot be predicted. All 3 complete circumferential cement mantle fractures were detected as a sudden and disproportionate increase in migration between 2 follow-up occasions. The fourth case did not involve a complete circumferential cement mantle fracture and was not accompanied by large migration. Although anecdotal due to the small number of stems with complete cement mantle fractures, this suggests that RSA may be able to predict long-term failure of implants designed to migrate: implants with cement fractures that are accompanied by sudden and disproportionally high migration are at risk of late loosening.

Since the cement mantle fractures and their corresponding disproportional increase in migration were noted within the first 3 years of follow-up, it may be necessary to follow prostheses designed to migrate—and thus continue to load the cement mantle—for more than the first 2 years with RSA, e.g. the first 4 or 5 years. This is because in implants designed to migrate in the cement mantle, compatibility of this design with the cement mantle (i.e. whether this is withstood by the cement mantle) is also of importance (Shen 1998).

A limitation of our study is that there were no markers implanted into the cement mantle. Thus, we were not able to determine whether the migration measured was migration in the prosthesis-cement interface or in the cement-bone interface. However, for the Exeter femoral stem it has been shown that migration of the prosthesis is relative to the cement mantle, which does not migrate relative to the bone (Alfaro-Adrian et al. 1999, McCalden et al. 2010). It is therefore safe to assume that the migration measured was relative to the cement mantle.

Although 41 THAs is an adequate sample size for short-term RSA follow-up, in combination with a high number of deaths during follow-up, this represents a limitation of the study. However, no patients were lost to follow-up. Another limitation is the fact that the study group might not be representative of the more general population: we had a high proportion of women and patients with rheumatoid arthritis due to fact that the study was performed in a tertiary referral center specialized in surgery in patients with rheumatoid arthritis. Also, due to the nature of the initial short-term study, two types of cement with different viscosity were used. However, neither the underlying pathology nor the cement type (viscosity) was found to influence migration to a statistically significant extent.

In conclusion, the Exeter femoral stem continues to migrate during the first 12 years after implantation. Since this femoral stem has excellent survivorship, this indicates that absolute stability is not required for good long-term outcome of implants that are designed to migrate during their lifetime.
